# Supplementary material for: Characterization of Viral miRNAs during Adenovirus 14 Infection and Their Differential Expression in the Emergent Strain Adenovirus 14p1
Source: Viruses. 2022 Apr 26;14(5):898. doi: 10.3390/v14050898 (PMC9145648; doi:10.3390/v14050898)
Supplement: Supplementary file 1 [file viruses-14-00898-s001.zip › viruses-1676449-supplementary-Table S3.pdf]

KEGG Results
Pathways (number of targets)

- hsa01100 Metabolic pathways - Homo sapiens (human) (141)
- hsa05200 Pathways in cancer - Homo sapiens (human) (73)
- hsa04151 PI3K-Akt signaling pathway - Homo sapiens (human) (49)
- hsa05022 Pathways of neurodegeneration - multiple diseases - Homo sapiens (human) (42)
- hsa05205 Proteoglycans in cancer - Homo sapiens (human) (41)
- hsa05168 Herpes simplex virus 1 infection - Homo sapiens (human) (40)
- hsa05165 Human papillomavirus infection - Homo sapiens (human) (40)
- hsa04010 MAPK signaling pathway - Homo sapiens (human) (37)
- hsa04810 Regulation of actin cytoskeleton - Homo sapiens (human) (36)
- hsa04024 cAMP signaling pathway - Homo sapiens (human) (35)
- hsa04360 Axon guidance - Homo sapiens (human) (33)
- hsa05224 Breast cancer - Homo sapiens (human) (32)
- hsa04015 Rap1 signaling pathway - Homo sapiens (human) (32)
- hsa04510 Focal adhesion - Homo sapiens (human) (32)
- hsa04150 mTOR signaling pathway - Homo sapiens (human) (31)
- hsa04020 Calcium signaling pathway - Homo sapiens (human) (31)
- hsa04014 Ras signaling pathway - Homo sapiens (human) (31)
- hsa04310 Wnt signaling pathway - Homo sapiens (human) (29)
- hsa05131 Shigellosis - Homo sapiens (human) (29)
- hsa04921 Oxytocin signaling pathway - Homo sapiens (human) (29)
- hsa05014 Amyotrophic lateral sclerosis - Homo sapiens (human) (28)
- hsa04934 Cushing syndrome - Homo sapiens (human) (28)
- hsa05010 Alzheimer disease - Homo sapiens (human) (28)
- hsa04144 Endocytosis - Homo sapiens (human) (28)
- hsa04530 Tight junction - Homo sapiens (human) (27)
- hsa05163 Human cytomegalovirus infection - Homo sapiens (human) (27)
- hsa04080 Neuroactive ligand-receptor interaction - Homo sapiens (human) (27)
- hsa04550 Signaling pathways regulating pluripotency of stem cells - Homo sapiens (human) (26)
- hsa04022 cGMP-PKG signaling pathway - Homo sapiens (human) (26)
- hsa05206 MicroRNAs in cancer - Homo sapiens (human) (25)
- hsa04390 Hippo signaling pathway - Homo sapiens (human) (25)
- hsa05202 Transcriptional misregulation in cancer - Homo sapiens (human) (25)
- hsa04270 Vascular smooth muscle contraction - Homo sapiens (human) (24)
- hsa04261 Adrenergic signaling in cardiomyocytes - Homo sapiens (human) (24)
- hsa05225 Hepatocellular carcinoma - Homo sapiens (human) (23)
- hsa04120 Ubiquitin mediated proteolysis - Homo sapiens (human) (23)
- hsa05161 Hepatitis B - Homo sapiens (human) (23)
- hsa04068 FoxO signaling pathway - Homo sapiens (human) (22)
- hsa04114 Oocyte meiosis - Homo sapiens (human) (22)
- hsa05170 Human immunodeficiency virus 1 infection - Homo sapiens (human) (22)

- hsa04152 AMPK signaling pathway - Homo sapiens (human) (22)
- hsa03040 Spliceosome - Homo sapiens (human) (22)
- hsa04722 Neurotrophin signaling pathway - Homo sapiens (human) (21)
- hsa04916 Melanogenesis - Homo sapiens (human) (21)
- hsa05226 Gastric cancer - Homo sapiens (human) (21)
- hsa04060 Cytokine-cytokine receptor interaction - Homo sapiens (human) (21)
- hsa04140 Autophagy - animal - Homo sapiens (human) (21)
- hsa04611 Platelet activation - Homo sapiens (human) (20)
- hsa04910 Insulin signaling pathway - Homo sapiens (human) (20)
- hsa05016 Huntington disease - Homo sapiens (human) (20)
- hsa04218 Cellular senescence - Homo sapiens (human) (20)
- hsa04071 Sphingolipid signaling pathway - Homo sapiens (human) (20)
- hsa04211 Longevity regulating pathway - Homo sapiens (human) (20)
- hsa05203 Viral carcinogenesis - Homo sapiens (human) (20)
- hsa05132 Salmonella infection - Homo sapiens (human) (20)
- hsa04072 Phospholipase D signaling pathway - Homo sapiens (human) (19)
- hsa05417 Lipid and atherosclerosis - Homo sapiens (human) (19)
- hsa03015 mRNA surveillance pathway - Homo sapiens (human) (19)
- hsa04750 Inflammatory mediator regulation of TRP channels - Homo sapiens (human) (19)
- hsa05130 Pathogenic Escherichia coli infection - Homo sapiens (human) (19)
- hsa04062 Chemokine signaling pathway - Homo sapiens (human) (19)
- hsa05160 Hepatitis C - Homo sapiens (human) (19)
- hsa04371 Apelin signaling pathway - Homo sapiens (human) (19)
- hsa04928 Parathyroid hormone synthesis, secretion and action - Homo sapiens (human) (18)
- hsa01522 Endocrine resistance - Homo sapiens (human) (18)
- hsa04141 Protein processing in endoplasmic reticulum - Homo sapiens (human) (18)
- hsa04925 Aldosterone synthesis and secretion - Homo sapiens (human) (18)
- hsa05418 Fluid shear stress and atherosclerosis - Homo sapiens (human) (18)
- hsa05166 Human T-cell leukemia virus 1 infection - Homo sapiens (human) (18)
- hsa04621 NOD-like receptor signaling pathway - Homo sapiens (human) (17)
- hsa04912 GnRH signaling pathway - Homo sapiens (human) (17)
- hsa04915 Estrogen signaling pathway - Homo sapiens (human) (17)
- hsa03013 RNA transport - Homo sapiens (human) (17)
- hsa05135 Yersinia infection - Homo sapiens (human) (17)
- hsa04728 Dopaminergic synapse - Homo sapiens (human) (17)
- hsa05167 Kaposi sarcoma-associated herpesvirus infection - Homo sapiens (human) (17)
- hsa04630 JAK-STAT signaling pathway - Homo sapiens (human) (17)
- hsa04724 Glutamatergic synapse - Homo sapiens (human) (17)
- hsa00230 Purine metabolism - Homo sapiens (human) (17)
- hsa05017 Spinocerebellar ataxia - Homo sapiens (human) (16)
- hsa05152 Tuberculosis - Homo sapiens (human) (16)
- hsa04350 TGF-beta signaling pathway - Homo sapiens (human) (16)

- hsa04714 Thermogenesis - Homo sapiens (human) (16)
- hsa04931 Insulin resistance - Homo sapiens (human) (16)
- hsa05231 Choline metabolism in cancer - Homo sapiens (human) (16)
- hsa04012 ErbB signaling pathway - Homo sapiens (human) (16)
- hsa04514 Cell adhesion molecules - Homo sapiens (human) (16)
- hsa04974 Protein digestion and absorption - Homo sapiens (human) (15)
- hsa04540 Gap junction - Homo sapiens (human) (15)
- hsa00564 Glycerophospholipid metabolism - Homo sapiens (human) (15)
- hsa04914 Progesterone-mediated oocyte maturation - Homo sapiens (human) (15)
- hsa05415 Diabetic cardiomyopathy - Homo sapiens (human) (15)
- hsa04725 Cholinergic synapse - Homo sapiens (human) (15)
- hsa05034 Alcoholism - Homo sapiens (human) (15)
- hsa05169 Epstein-Barr virus infection - Homo sapiens (human) (15)
- hsa05414 Dilated cardiomyopathy - Homo sapiens (human) (15)
- hsa04935 Growth hormone synthesis, secretion and action - Homo sapiens (human) (15)
- hsa04670 Leukocyte transendothelial migration - Homo sapiens (human) (15)
- hsa04666 Fc gamma R-mediated phagocytosis - Homo sapiens (human) (14)
- hsa04217 Necroptosis - Homo sapiens (human) (14)
- hsa04520 Adherens junction - Homo sapiens (human) (14)
- hsa05215 Prostate cancer - Homo sapiens (human) (14)
- hsa04919 Thyroid hormone signaling pathway - Homo sapiens (human) (14)
- hsa05410 Hypertrophic cardiomyopathy - Homo sapiens (human) (14)
- hsa04971 Gastric acid secretion - Homo sapiens (human) (14)
- hsa01521 EGFR tyrosine kinase inhibitor resistance - Homo sapiens (human) (14)
- hsa04720 Long-term potentiation - Homo sapiens (human) (14)
- hsa04213 Longevity regulating pathway - multiple species - Homo sapiens (human) (14)
- hsa05214 Glioma - Homo sapiens (human) (14)
- hsa05012 Parkinson disease - Homo sapiens (human) (13)
- hsa04070 Phosphatidylinositol signaling system - Homo sapiens (human) (13)
- hsa04066 HIF-1 signaling pathway - Homo sapiens (human) (13)
- hsa05218 Melanoma - Homo sapiens (human) (13)
- hsa04110 Cell cycle - Homo sapiens (human) (13)
- hsa04926 Relaxin signaling pathway - Homo sapiens (human) (13)
- hsa04730 Long-term depression - Homo sapiens (human) (13)
- hsa05217 Basal cell carcinoma - Homo sapiens (human) (13)
- hsa05020 Prion disease - Homo sapiens (human) (13)
- hsa00514 Other types of O-glycan biosynthesis - Homo sapiens (human) (13)
- hsa04713 Circadian entrainment - Homo sapiens (human) (13)
- hsa04668 TNF signaling pathway - Homo sapiens (human) (13)
- hsa04723 Retrograde endocannabinoid signaling - Homo sapiens (human) (13)
- hsa05235 PD-L1 expression and PD-1 checkpoint pathway in cancer - Homo sapiens (human) (12)
- hsa04622 RIG-I-like receptor signaling pathway - Homo sapiens (human) (12)
- hsa04924 Renin secretion - Homo sapiens (human) (12)

- hsa03018 RNA degradation - Homo sapiens (human) (12)
- hsa04380 Osteoclast differentiation - Homo sapiens (human) (12)
- hsa05171 Coronavirus disease - COVID-19 - Homo sapiens (human) (12)
- hsa04970 Salivary secretion - Homo sapiens (human) (11)
- hsa04932 Non-alcoholic fatty liver disease - Homo sapiens (human) (11)
- hsa04137 Mitophagy - animal - Homo sapiens (human) (11)
- hsa04978 Mineral absorption - Homo sapiens (human) (11)
- hsa05222 Small cell lung cancer - Homo sapiens (human) (11)
- hsa04972 Pancreatic secretion - Homo sapiens (human) (11)
- hsa04922 Glucagon signaling pathway - Homo sapiens (human) (11)
- hsa05162 Measles - Homo sapiens (human) (11)
- hsa04726 Serotonergic synapse - Homo sapiens (human) (11)
- hsa05031 Amphetamine addiction - Homo sapiens (human) (11)
- hsa05100 Bacterial invasion of epithelial cells - Homo sapiens (human) (11)
- hsa04625 C-type lectin receptor signaling pathway - Homo sapiens (human) (11)
- hsa00510 N-Glycan biosynthesis - Homo sapiens (human) (11)
- hsa04917 Prolactin signaling pathway - Homo sapiens (human) (11)
- hsa01240 Biosynthesis of cofactors - Homo sapiens (human) (11)
- hsa04142 Lysosome - Homo sapiens (human) (10)
- hsa04660 T cell receptor signaling pathway - Homo sapiens (human) (10)
- hsa00565 Ether lipid metabolism - Homo sapiens (human) (10)
- hsa03460 Fanconi anemia pathway - Homo sapiens (human) (10)
- hsa05210 Colorectal cancer - Homo sapiens (human) (10)
- hsa04145 Phagosome - Homo sapiens (human) (10)
- hsa05146 Amoebiasis - Homo sapiens (human) (10)
- hsa04620 Toll-like receptor signaling pathway - Homo sapiens (human) (10)
- hsa04210 Apoptosis - Homo sapiens (human) (10)
- hsa04613 Neutrophil extracellular trap formation - Homo sapiens (human) (10)
- hsa05032 Morphine addiction - Homo sapiens (human) (10)
- hsa04115 p53 signaling pathway - Homo sapiens (human) (10)
- hsa04721 Synaptic vesicle cycle - Homo sapiens (human) (10)
- hsa05412 Arrhythmogenic right ventricular cardiomyopathy - Homo sapiens (human) (9)
- hsa04740 Olfactory transduction - Homo sapiens (human) (9)
- hsa04923 Regulation of lipolysis in adipocytes - Homo sapiens (human) (9)
- hsa04933 AGE-RAGE signaling pathway in diabetic complications - Homo sapiens (human) (9)
- hsa04340 Hedgehog signaling pathway - Homo sapiens (human) (9)
- hsa04650 Natural killer cell mediated cytotoxicity - Homo sapiens (human) (9)
- hsa05142 Chagas disease - Homo sapiens (human) (9)
- hsa05221 Acute myeloid leukemia - Homo sapiens (human) (9)
- hsa04727 GABAergic synapse - Homo sapiens (human) (9)
- hsa04658 Th1 and Th2 cell differentiation - Homo sapiens (human) (9)
- hsa04330 Notch signaling pathway - Homo sapiens (human) (9)
- hsa04064 NF-kappa B signaling pathway - Homo sapiens (human) (9)

- hsa05220 Chronic myeloid leukemia - Homo sapiens (human) (9)
- hsa04662 B cell receptor signaling pathway - Homo sapiens (human) (8)
- hsa04961 Endocrine and other factor-regulated calcium reabsorption - Homo sapiens (human) (8)
- hsa05212 Pancreatic cancer - Homo sapiens (human) (8)
- hsa04927 Cortisol synthesis and secretion - Homo sapiens (human) (8)
- hsa01200 Carbon metabolism - Homo sapiens (human) (8)
- hsa04659 Th17 cell differentiation - Homo sapiens (human) (8)
- hsa04913 Ovarian steroidogenesis - Homo sapiens (human) (8)
- hsa04260 Cardiac muscle contraction - Homo sapiens (human) (8)
- hsa05211 Renal cell carcinoma - Homo sapiens (human) (8)
- hsa04911 Insulin secretion - Homo sapiens (human) (8)
- hsa04512 ECM-receptor interaction - Homo sapiens (human) (8)
- hsa05223 Non-small cell lung cancer - Homo sapiens (human) (8)
- hsa04710 Circadian rhythm - Homo sapiens (human) (8)
- hsa00600 Sphingolipid metabolism - Homo sapiens (human) (8)
- hsa04664 Fc epsilon RI signaling pathway - Homo sapiens (human) (7)
- hsa00513 Various types of N-glycan biosynthesis - Homo sapiens (human) (7)
- hsa04920 Adipocytokine signaling pathway - Homo sapiens (human) (7)
- hsa05120 Epithelial cell signaling in Helicobacter pylori infection - Homo sapiens (human) (7)
- hsa05213 Endometrial cancer - Homo sapiens (human) (7)
- hsa05133 Pertussis - Homo sapiens (human) (7)
- hsa04146 Peroxisome - Homo sapiens (human) (7)
- hsa05030 Cocaine addiction - Homo sapiens (human) (7)
- hsa00240 Pyrimidine metabolism - Homo sapiens (human) (7)
- hsa04918 Thyroid hormone synthesis - Homo sapiens (human) (7)
- hsa05145 Toxoplasmosis - Homo sapiens (human) (7)
- hsa04930 Type II diabetes mellitus - Homo sapiens (human) (7)
- hsa05164 Influenza A - Homo sapiens (human) (7)
- hsa04610 Complement and coagulation cascades - Homo sapiens (human) (7)
- hsa00590 Arachidonic acid metabolism - Homo sapiens (human) (7)
- hsa01524 Platinum drug resistance - Homo sapiens (human) (7)
- hsa05134 Legionellosis - Homo sapiens (human) (6)
- hsa04976 Bile secretion - Homo sapiens (human) (6)
- hsa04061 Viral protein interaction with cytokine and cytokine receptor - Homo sapiens (human) (6)
- hsa05110 Vibrio cholerae infection - Homo sapiens (human) (6)
- hsa01230 Biosynthesis of amino acids - Homo sapiens (human) (6)
- hsa05323 Rheumatoid arthritis - Homo sapiens (human) (6)
- hsa05321 Inflammatory bowel disease - Homo sapiens (human) (6)
- hsa04640 Hematopoietic cell lineage - Homo sapiens (human) (6)
- hsa00512 Mucin type O-glycan biosynthesis - Homo sapiens (human) (6)
- hsa04960 Aldosterone-regulated sodium reabsorption - Homo sapiens (human) (5)

- hsa03440 Homologous recombination - Homo sapiens (human) (5)
- hsa04973 Carbohydrate digestion and absorption - Homo sapiens (human) (5)
- hsa04657 IL-17 signaling pathway - Homo sapiens (human) (5)
- hsa00520 Amino sugar and nucleotide sugar metabolism - Homo sapiens (human) (5)
- hsa00562 Inositol phosphate metabolism - Homo sapiens (human) (5)
- hsa00983 Drug metabolism - other enzymes - Homo sapiens (human) (5)
- hsa05230 Central carbon metabolism in cancer - Homo sapiens (human) (5)
- hsa00592 alpha-Linolenic acid metabolism - Homo sapiens (human) (5)
- hsa04370 VEGF signaling pathway - Homo sapiens (human) (5)
- hsa03022 Basal transcription factors - Homo sapiens (human) (5)
- hsa05140 Leishmaniasis - Homo sapiens (human) (5)
- hsa04623 Cytosolic DNA-sensing pathway - Homo sapiens (human) (5)
- hsa00270 Cysteine and methionine metabolism - Homo sapiens (human) (5)
- hsa01040 Biosynthesis of unsaturated fatty acids - Homo sapiens (human) (5)
- hsa00310 Lysine degradation - Homo sapiens (human) (5)
- hsa00190 Oxidative phosphorylation - Homo sapiens (human) (5)
- hsa04136 Autophagy - other - Homo sapiens (human) (5)
- hsa00760 Nicotinate and nicotinamide metabolism - Homo sapiens (human) (5)
- hsa05219 Bladder cancer - Homo sapiens (human) (4)
- hsa04962 Vasopressin-regulated water reabsorption - Homo sapiens (human) (4)
- hsa05216 Thyroid cancer - Homo sapiens (human) (4)
- hsa00260 Glycine, serine and threonine metabolism - Homo sapiens (human) (4)
- hsa04929 GnRH secretion - Homo sapiens (human) (4)
- hsa01212 Fatty acid metabolism - Homo sapiens (human) (4)
- hsa00480 Glutathione metabolism - Homo sapiens (human) (4)
- hsa00982 Drug metabolism - cytochrome P450 - Homo sapiens (human) (4)
- hsa04130 SNARE interactions in vesicular transport - Homo sapiens (human) (4)
- hsa00604 Glycosphingolipid biosynthesis - ganglio series - Homo sapiens (human) (4)
- hsa03008 Ribosome biogenesis in eukaryotes - Homo sapiens (human) (4)
- hsa00591 Linoleic acid metabolism - Homo sapiens (human) (4)
- hsa05033 Nicotine addiction - Homo sapiens (human) (4)
- hsa00561 Glycerolipid metabolism - Homo sapiens (human) (4)
- hsa00534 Glycosaminoglycan biosynthesis - heparan sulfate / heparin - Homo sapiens (human) (4)
- hsa00010 Glycolysis / Gluconeogenesis - Homo sapiens (human) (4)
- hsa00051 Fructose and mannose metabolism - Homo sapiens (human) (4)
- hsa04614 Renin-angiotensin system - Homo sapiens (human) (3)
- hsa04966 Collecting duct acid secretion - Homo sapiens (human) (3)
- hsa04742 Taste transduction - Homo sapiens (human) (3)
- hsa00563 Glycosylphosphatidylinositol (GPI)-anchor biosynthesis - Homo sapiens (human) (3)
- hsa03420 Nucleotide excision repair - Homo sapiens (human) (3)
- hsa03050 Proteasome - Homo sapiens (human) (3)
- hsa01523 Antifolate resistance - Homo sapiens (human) (3)

- hsa00515 Mannose type O-glycan biosynthesis - Homo sapiens (human) (3)
- hsa00770 Pantothenate and CoA biosynthesis - Homo sapiens (human) (3)
- hsa05204 Chemical carcinogenesis - Homo sapiens (human) (3)
- hsa00440 Phosphonate and phosphinate metabolism - Homo sapiens (human) (3)
- hsa00250 Alanine, aspartate and glutamate metabolism - Homo sapiens (human) (3)
- hsa00740 Riboflavin metabolism - Homo sapiens (human) (3)
- hsa04612 Antigen processing and presentation - Homo sapiens (human) (3)
- hsa00380 Tryptophan metabolism - Homo sapiens (human) (3)
- hsa00980 Metabolism of xenobiotics by cytochrome P450 - Homo sapiens (human) (3)
- hsa00052 Galactose metabolism - Homo sapiens (human) (3)
- hsa00280 Valine, leucine and isoleucine degradation - Homo sapiens (human) (3)
- hsa00500 Starch and sucrose metabolism - Homo sapiens (human) (3)
- hsa03450 Non-homologous end-joining - Homo sapiens (human) (3)
- hsa04975 Fat digestion and absorption - Homo sapiens (human) (3)
- hsa00062 Fatty acid elongation - Homo sapiens (human) (3)
- hsa00071 Fatty acid degradation - Homo sapiens (human) (3)
- hsa04977 Vitamin digestion and absorption - Homo sapiens (human) (3)
- hsa04950 Maturity onset diabetes of the young - Homo sapiens (human) (3)
- hsa04216 Ferroptosis - Homo sapiens (human) (3)
- hsa03010 Ribosome - Homo sapiens (human) (3)
- hsa03020 RNA polymerase - Homo sapiens (human) (3)
- hsa00601 Glycosphingolipid biosynthesis - lacto and neolacto series - Homo sapiens (human) (3)
- hsa00511 Other glycan degradation - Homo sapiens (human) (2)
- hsa04672 Intestinal immune network for IgA production - Homo sapiens (human) (2)
- hsa00830 Retinol metabolism - Homo sapiens (human) (2)
- hsa00120 Primary bile acid biosynthesis - Homo sapiens (human) (2)
- hsa05150 Staphylococcus aureus infection - Homo sapiens (human) (2)
- hsa04392 Hippo signaling pathway - multiple species - Homo sapiens (human) (2)
- hsa00220 Arginine biosynthesis - Homo sapiens (human) (2)
- hsa00730 Thiamine metabolism - Homo sapiens (human) (2)
- hsa05310 Asthma - Homo sapiens (human) (2)
- hsa02010 ABC transporters - Homo sapiens (human) (2)
- hsa03060 Protein export - Homo sapiens (human) (2)
- hsa01210 2-Oxocarboxylic acid metabolism - Homo sapiens (human) (2)
- hsa00330 Arginine and proline metabolism - Homo sapiens (human) (2)
- hsa00360 Phenylalanine metabolism - Homo sapiens (human) (2)
- hsa00620 Pyruvate metabolism - Homo sapiens (human) (2)
- hsa04979 Cholesterol metabolism - Homo sapiens (human) (2)
- hsa00533 Glycosaminoglycan biosynthesis - keratan sulfate - Homo sapiens (human) (2)
- hsa00130 Ubiquinone and other terpenoid-quinone biosynthesis - Homo sapiens (human) (2)
- hsa00100 Steroid biosynthesis - Homo sapiens (human) (2)
- hsa00790 Folate biosynthesis - Homo sapiens (human) (2)

- hsa05340 Primary immunodeficiency - Homo sapiens (human) (2)
- hsa00670 One carbon pool by folate - Homo sapiens (human) (2)
- hsa00430 Taurine and hypotaurine metabolism - Homo sapiens (human) (2)
- hsa03320 PPAR signaling pathway - Homo sapiens (human) (2)
- hsa05322 Systemic lupus erythematosus - Homo sapiens (human) (2)
- hsa03410 Base excision repair - Homo sapiens (human) (2)
- hsa00900 Terpenoid backbone biosynthesis - Homo sapiens (human) (2)
- hsa00350 Tyrosine metabolism - Homo sapiens (human) (2)
- hsa00140 Steroid hormone biosynthesis - Homo sapiens (human) (2)
- hsa05416 Viral myocarditis - Homo sapiens (human) (2)
- hsa05320 Autoimmune thyroid disease - Homo sapiens (human) (2)
- hsa04744 Phototransduction - Homo sapiens (human) (2)
- hsa00860 Porphyrin and chlorophyll metabolism - Homo sapiens (human) (2)
- hsa05144 Malaria - Homo sapiens (human) (1)
- hsa00340 Histidine metabolism - Homo sapiens (human) (1)
- hsa00450 Selenocompound metabolism - Homo sapiens (human) (1)
- hsa00290 Valine, leucine and isoleucine biosynthesis - Homo sapiens (human) (1)
- hsa04964 Proximal tubule bicarbonate reclamation - Homo sapiens (human) (1)
- hsa00030 Pentose phosphate pathway - Homo sapiens (human) (1)
- hsa05143 African trypanosomiasis - Homo sapiens (human) (1)
- hsa00640 Propanoate metabolism - Homo sapiens (human) (1)
- hsa05330 Allograft rejection - Homo sapiens (human) (1)
- hsa04940 Type I diabetes mellitus - Homo sapiens (human) (1)
- hsa05332 Graft-versus-host disease - Homo sapiens (human) (1)
- hsa00630 Glyoxylate and dicarboxylate metabolism - Homo sapiens (human) (1)
- hsa04215 Apoptosis - multiple species - Homo sapiens (human) (1)
- hsa03030 DNA replication - Homo sapiens (human) (1)
- hsa00910 Nitrogen metabolism - Homo sapiens (human) (1)
- hsa00410 beta-Alanine metabolism - Homo sapiens (human) (1)
- hsa00785 Lipoic acid metabolism - Homo sapiens (human) (1)
- hsa00524 Neomycin, kanamycin and gentamicin biosynthesis - Homo sapiens (human) (1)
- hsa00532 Glycosaminoglycan biosynthesis - chondroitin sulfate / dermatan sulfate - Homo sapiens (human) (1)
- hsa00970 Aminoacyl-tRNA biosynthesis - Homo sapiens (human) (1)
- hsa00531 Glycosaminoglycan degradation - Homo sapiens (human) (1)
- hsa00603 Glycosphingolipid biosynthesis - globo and isoglobo series - Homo sapiens (human) (1)

Brite

hsa00001 KEGG Orthology (KO) (75)

- hsa03000 Transcription factors (51)
- hsa04121 Ubiquitin system (7)

- hsa01000 Enzymes (5)
- hsa03041 Spliceosome (4)
- hsa03400 DNA repair and recombination proteins (4)
- hsa04990 Domain-containing proteins not elsewhere classified (4)
- hsa03036 Chromosome and associated proteins (4)
- hsa04147 Exosome (4)
- hsa01009 Protein phosphatases and associated proteins (2)
- hsa04131 Membrane trafficking (1)
- hsa03019 Messenger RNA biogenesis (1)
- hsa03021 Transcription machinery (1)
- hsa03029 Mitochondrial biogenesis (1)
